# Fontan Circulation and Aortic Stiffness: Insights into Vascular Dynamics and Haemodynamic Interplay

**DOI:** 10.1007/s00246-024-03572-z

**Published:** 2024-07-15

**Authors:** Matthias Walser, Leonie Arnold, Guido Mandilaras, Christoph Funk, Robert Dalla-Pozza, Joseph Pattathu, Nikolaus A. Haas, André Jakob

**Affiliations:** Department of Paediatric Cardiology and Paediatric Intensive Care, Ludwig-Maximilians-University of Munich, Marchioninistr. 15, 81377 Munich, Germany

**Keywords:** Pulse wave velocity (PWV), Fontan, Single ventricle, Aortic stiffness, Haemodynamics

## Abstract

**Abstract:**

Increased aortic stiffness predisposes cardiac afterload and influences cardiac function. Congenital heart diseases involving aortic arch malformation and extended cardiovascular surgery, i.e. univentricular heart diseases, can lead to increased aortic stiffness. This study aimed to investigate whether Fontan patients (FO) have increased aortic stiffness within distinct aortic segments, and whether these parameters relate to Fontan-specific haemodynamics. In a prospective case–control study, 20 FO and 49 heart-transplanted control subjects with biventricular circulation underwent invasive cardiac catheterisation. We invasively measured pulse wave velocity (PWV) in the ascending aorta and along the entire aorta. Haemodynamic parameters, including end-diastolic pressure, pulmonary artery pressure, the cardiac index and systemic vascular resistance index were also assessed. FO exhibited significantly higher ascending aorta PWV (aPWV) than controls (FO: 7.2 ± 2.4 m/s|Controls: 4.9 ± 0.7 m/s, *p* < 0.001) and compared to the inner group central aorta PWV (cPWV; FO: 5.5 ± 1.2 m/s|Controls: 5.3 ± 1.0 m/s). Multivariate analysis confirmed this aPWV elevation in FO even after adjusting for age and BMI. aPWV and cPWV were almost identical within the control group. Correlation analyses revealed associations between cPWV and blood pressure in controls, while correlations were less apparent in FO. We detected no significant association between the aPWV and other haemodynamic parameters in any of our groups. FO exhibit increased aPWV, indicating specific vascular stiffness in the ascending aorta, while their overall aortic stiffness remains comparable to controls. Further research is needed to understand the implications of these findings on Fontan circulation and long-term cardiovascular health.

**Central Message:**

Fontan patients show increased aortic arch pulse wave velocity, suggesting specific vascular stiffness.

**Perspective Statement:**

Our study offers rare insights into pulse wave velocity in Fontan patients, highlighting increased arterial stiffness in the aortic arch. Vascular stiffness was particularly increased in the area of surgical reconstruction. This indicates the need for further research on vascular stiffness in Fontan circulation to understand its impact on cardiovascular health.

**Clinical Trial Registration:**

German clinical trial registration, DRKS00015066.

## Introduction

Different complex congenital heart diseases, mainly those involving single ventricular anatomy (SiV), are unable to undergo biventricular repair. To separate systemic and pulmonary circulation in these patients, several procedures are necessary, with the last commonly known as the Fontan operation. Morbidity and mortality have decreased as the treatment of Fontan patients (FO) continues to improve, and more patients are now reaching adulthood [1]. Nevertheless, these patients face specific physiological alterations that have long-term consequences. Predominantly, without a pumping subpulmonary ventricle, the caval veins are directly connected to the pulmonary arteries. On the one hand, this passive blood flow through the pulmonary vascular bed leads to upstream congestion and elevated systemic venous pressure [2]. The resulting continuous venous “backlog” leads, via rising central venous pressures, to organ disorders such as liver cirrhosis [1], protein-losing enteropathy and plastic bronchitis [3]. On the other hand, diminished downstream blood flow to the systemic ventricle also influences cardiac function. Decreased cardiac output (CO) as a result of reduced backflow is followed by arterial vasoconstriction, and thus an increase in afterload [2, 4, 5]. This increased afterload results in abnormal ventricular relaxation and reduced contractility [5–7]. In addition, complex surgical aortic arch reconstructions are part of many surgical palliative procedures in SiV. Direct effects on the arterial vessels, e.g., arterial wall remodelling with increased thickness leading to reduced elasticity and greater vascular stiffness [8–11], may further impact Fontan haemodynamics [12, 13].

In the following prospective case–control study, we took invasive measurements to determine pulse wave velocity (PWV) in patients with Fontan circulation and compared those with measurements from control subjects with biventricular physiology. PWV was measured at the main site of surgical manipulation in the ascending aorta and at the entire aorta. We then investigated whether potentially altered vascular stiffness has an impact on other haemodynamics such as end-diastolic pressure (EDP), pulmonary artery pressure (PA), the cardiac index (CI) or sytemic vascular resistance index (SVRI).

## Methods

### Study Population

Our Fontan patients (FO) group who underwent diagnostic or interventional cardiac catheterisation were recruited prospectively between 2019 and 2022. As for our control-group, we recruited heart transplant patients presenting a normal biventricular cardiac anatomy and function, who also underwent routine cardiac catheterisation. All cardiac catheterisations were performed at the Department of Paediatric Cardiology and Intensive Care, Ludwig-Maximilians-University of Munich. Written informed consent was required from the patient and/or parents/legal guardians before participation in the study.

Exclusion criteria for our control group were any sign of flow obstruction, of congestive heart failure or cardiac arrhythmia. In FO, only patients who had undergone the complete Fontan procedure were included, presenting no signs of systemic outflow obstructions. Approval for this study was provided by the Ethics Committee of Ludwig-Maximilians-University of Munich in accordance with the ethical standards of the Declaration of Helsinki (Vote Number: 18-816).

### Data Acquisition

Cardiac catheterisation was conducted in all patients under analgosedation with midazolam, ketamine, and propofol as required. A 4–5 French, fluid-filled multipurpose catheter (MP) (Cordis, MPA 2 SH, 125 cm, EMEA, Dublin, Ireland) was introduced over a 5–6 French femoral artery sheath (Terumo, Radifocus Introducer II, Leuven, Belgium). We relied on the phlebostatic axis to zero in on the haemodynamic measurements. Catheter positions were assessed fluoroscopically and all recorded values were digitally documented and stored for further analysis (Sensis, Siemens Healthcare, Erlangen, Germany).

We have previously reported on our methodology taking invasive PWV measurements with adequate interobserver variability [14]. Briefly: PWV measurements were taken by documenting the travel distance and travel time, and then calculating the PWV applying the formula “velocity = distance/time.” We determined two PWVs: one for the entire central main artery spanning from the ascending aorta to the femoral artery (cPWV), and the other specifically for the transition time of the ascending aorta to aortic arch, representing the main site of aortic surgical manipulation (aPWV).

**Fig. 1:**
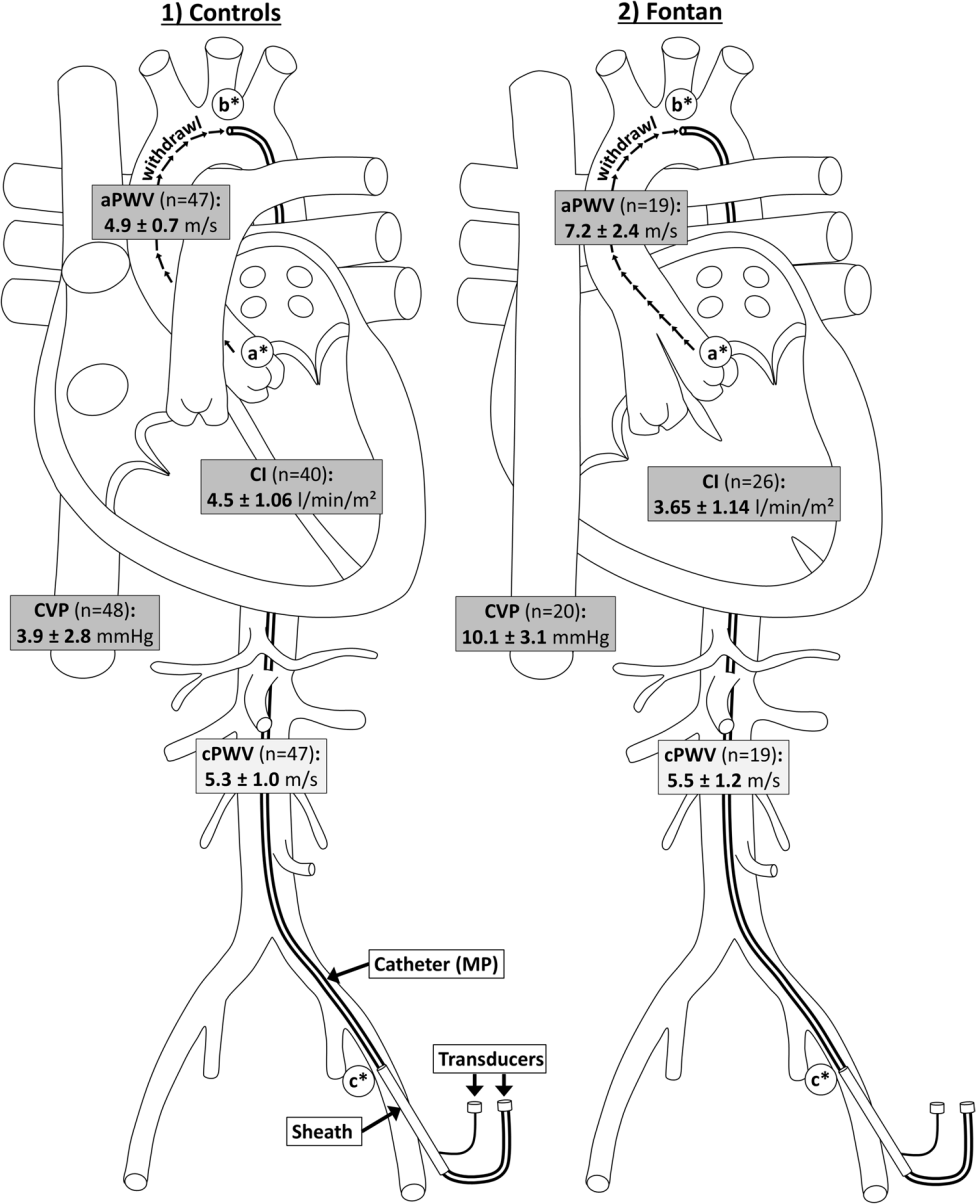
Schematic illustration of PWV methodology. Schematic illustration of PWV measurement methodology in both groups (1 = Controls, 2 = Fontan) with some major results. Different Measurement positions of the catheter are marked with a*–c*. Central venous pressure (CVP), Cardiac Index (CI) and pulse wave velocities of ascending (aPWV) and central aorta (cPWV) are presented ± standard derivation. Significant differences (*p* < 0.05), calculated using T-Test/Wilcoxon-rank-sum test, between values of both groups (Controls vs. Fontan) are highlighted in darker grey. PWV determination in ascending aorta (aPWV): The travel time was determined from the time difference between two measurements of pulse waves after withdrawing the catheter (→ → →) from the ascending aorta (a*) into the aortic arch (b*). The distance was calculated based on the length of catheter withdrawl. PWV determination of central aorta (cPWV): The travel time was determined from the time difference between two measurements of pulse waves between the ascending aorta (a*) and femoral artery (c*). The travel distance was determined from the catheter tip to the tip of the sheath

The MP catheter was first positioned in the ascending aorta/. (Fig. 1, a*) and then in the distal aortic arch. (Fig. 1, b*). PWV was documented simultaneously at the sheath tip within the iliac artery (Fig. 1, c*). Marking the catheters at the sheaths’ exit at both positions enabled us to later measure the travel distance. The travel time was calculated by measuring the distance digitally between the deepest point (footprint) of simultaneous MP and sheaths PWV readings together with the ECG at a recording speed of 200 mm/s.

Additional haemodynamic values were routinely obtained during catheterisation. In so doing, we determined central systolic blood pressure (SBP), diastolic blood pressure (DBP) and mean arterial blood pressure (MAP) in the ascending aorta (see Fig. 1). EDP, PAP, pulmonary capillary wedge pressure (PCWP), and central venous pressure (CVP) were also recorded whenever medically appropriate by considering the respective pulmonary arterial pressures of FO equal to the CVP of control patients, assuming there was no obstruction within the Fontan circulation.

Moreover, cardiac output (CO) and the cardiac index (CI) were calculated according to the well-known Fick-principle with the body surface area calculated via the Du Bois-formula. Here we also calculated systemic vascular resistance (SVR) and its index (SVRI).

### Data Analysis

Statistical analyses and plots were performed using R-Studio version 4.1.3 (R-Studio, Boston, USA). For descriptive analyses, we calculated measures of central tendency (mean) and dispersion (standard deviation, SD). Measurement results from our FO group were compared to those from the control group by dependent groups with the distribution evaluated via the Shapiro–Wilk Test. The groups were then analyzed using paired t-test for parametric and Wilcoxon-rank-sum test for non-parametric dependent samples. We also ran, a Pearson rank correlation analysis between PWV values and any of the parameters examined. In addition, a multivariate linear regression model was used to determine the influence of independent variables (Group, SBP, DBP, CI, CVP, EDP) on PWV as dependent variable. A *p* value ≤ 0.05 was considered statistically significant.

## Results

### Patients

Overall, 20 FO (9 male) with a mean-age of 18.1 ± 9.2 years (range 6–44 years) were compared to 49 control patients (23 male) with a mean-age of 18.5 ± 7.7 years (range 3–35 years). Patients’ baseline characteristics are listed in Table 1. The controls were mostly transplanted for cardiomyopathy. Five patients had hypoplastic left heart syndrome (HLHS). They were transplanted primarily (< 1 year) rather than secondarily due to failure of palliation. The FO group consisted of a very heterogeneous cohort with HLHS forming the largest group (*n* = 11) 0.18 FO patients received a Norwood operation with aortic arch reconstruction. The underlying congenital heart diseases of FO and controls are shown in Table 2. Both groups were equal in age, weight, and height, respectively, BMI with no significant difference. A relevant number of individuals in both groups were taking statins and on antihypertensive therapy consisting of ACE-inhibitors, calcium antagonists, beta blockers, diuretics, or a combined treatment. 37 control patients (76%) were on statins, while only 1 FO patient was (5%). On antihypertensive therapy were 42 control patients (86%) and 12 FO (60%). Applying paired t-test and Wilcoxon rank-sum test, we failed to identify any significant association between antihypertensive therapy and PWV in either FO or controls.

**Table 1:** Patients' profile (*n* = 69)

n	Fontan	Controls	*p* value
	20	49	
Male	9 (45%)	23 (47%)	
Age [years]	18.1 (± 9.2)	18.5 (± 7.7)	0.51
Height [cm]	156.2 (± 20.0)	158.5 (± 22.6)	0.51
Weight [kg]	48.3 (± 17.6)	53.2 (± 20.8)	0.33
BMI [kg/m^2^]	19.2 (± 4.0)	20.2 (± 4.5)	0.42
*Antihypertensive therapy*	12 (60%)	42 (86%)	
ACE-inhibitors	6	35	
Calcium antagonists	0	23	
Beta blockers	3	5	
Diuretics	10	8	
Statins	1 (5%)	37 (76%)	

Demographics and medications of all patients separated in Fontan-Patients and Control group (heart-transplanted patients). *P* values were calculated using paired T-Test for parametric and Wilcoxon-rank-sum test for non-parametric dependent samples. Antihypertensive therapy contains up to four drugs given in a combination of treatment. Data presented as mean (± SD) or n (%)

**Table 2:** Patients’ underlying diseases

Fontans' diseases (*n* = 20)	*n*
Hypoplastic left heart syndrome (HLHS)	11
Tricuspid atresia (TA)	3
Double inlet left ventricle (DILV)	2
Double outlet right ventricle (DORV) with uncommitted ventricular septal defect (VSD)	1
Unbalanced atrioventricular septal defect (AVSD)	1
Double inlet right ventricle (DIRV) with DORV + HLHS	1
Criss-cross-heart + VSD	1

Controls' diseases (*n* = 49)	*n*
Dilated cardiomyopathy	35
Hypoplastic left heart syndrome	5
Restrictive cardiomyopathy	3
Kawasaki syndrome	2
Ischemic cardiomyopathy	1
Chronic viral myocarditis	1
D-TGA with ventricular septal defect and pulmonary stenosis	1
Bland-White-Garland syndrome	1

Description of underlying heterogeneous diseases of our Fontan patients

### PWV

**Fig. 2:**
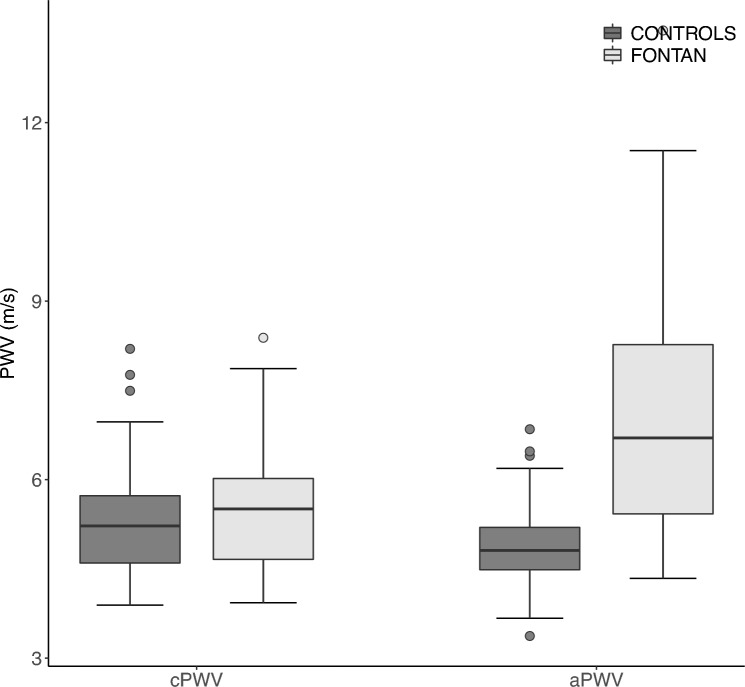
Boxplots showing mean PWV values in m/s, of Fontan- (*n* = 20) and Control-group (*n* = 49). *aPWV* pulse wave velocity of ascending aorta; *cPWV* pulse wave velocity of entire central aorta

Mean aPWV was significantly faster in FO than in controls (7.2 ± 2.4|4.9 ± 0.7 m/s; *p* < 0.001). Also, according to multivariate linear regression analysis adjusting for age and BMI, the aPWV was 2.5 m/s faster in FO (*p* = 0.003). Mean cPWVs were almost identical between the two groups (FO: 5.5 ± 1.2|Controls: 5.3 ± 1.0 m/s; *p* = 0.4), see Fig. 2. Table 3 illustrates our results from all measured PWVs and haemodynamic values in both groups.

**Table 3:** Measurement results

	All patients		Fontan		Controls		*p* value
	*n*		*n*		*n*		
aPWV [m/s]	5.6 ± 1.7	(66)	7.2 ± 2.4	(19)	4.9 ± 0.7	(47)	< 0.001*
cPWV [m/s]	5.3 ± 1.0	(66)	5.5 ± 1.2	(19)	5.3 ± 1.0	(47)	0.388
Systole [mmHg]	95.2 ± 18.2	(64)	90.8 ± 16.4	(20)	97.2 ± 18.8	(44)	0.289
Diastole [mmHg]	59.7 ± 11.4	(64)	59.6 ± 10.5	(20)	59.7 ± 12.0	(44)	0.971
MAP [mmHg]	74.5 ± 13.6	(64)	77.8 ± 12.3	(20)	75.7 ± 14.2	(44)	0.282
CVP [mmHg]	5.7 ± 4.1	(68)	10.1 ± 3.1	(20)	3.9 ± 2.8	(48)	< 0.001*
EDP [mmHg]	9.1 ± 3.6	(64)	8.4 ± 2.8	(19)	9.4 ± 3.8	(45)	0.593
PCWP [mmHg]	8.6 ± 3.4	(23)	7.7 ± 3.1	(24)	9.9 ± 3.6	(9)	0.155
CO [l/min]	6.38 ± 2.38	(56)	5.47 ± 2.59	(26)	6.75 ± 2.22	(40)	0.056
CI [l/min/m^2^]	4.26 ± 1.14	(56)	3.65 ± 1.14	(26)	4.50 ± 1.06	(40)	0.017*
SVR [dyn*sec/cm^5^]	974.9 ± 395.9	(49)	1069.6 ± 513.5	(15)	933.2 ± 332.1	(34)	0.4944
SVRI [dyn*s/cm^5^*m^2^]	1376.1 ± 457.5	(49)	1472.2 ± 576.8	(15)	1333.7 ± 396.5	(34)	0.3796

Values representing the respective mean with standard deviation (SD) and *p* values of T-Test test for parametric and Wilcoxon-rank-sum-test test for non-parametric dependent samples

*aPWV* Pulse wave velocity in ascending aorta, *cPWV* Pulse wave velocity in entire central aorta, *CI* cardiac index, *CO* cardiac output, *CVP* central venous pressure, *EDP* end-diastolic blood pressure, *MAD* mean arterial blood pressure, *PCWP* pulmonary capillary wedge pressure, *SVR* systemic vascular resistance, *SVRI* systemic vascular resistance index

Data given as mean (± SD). Significant results (*p* < 0.05) are marked with *

When comparing aPWV with cPWV within one group, we noted that aPWV was significantly faster than cPWV in the FO group (*p* = 0.009), whereas the two PWVs within the control group did not differ significantly (*p* = 0.1) (see Table 4).

**Table 4:** PWV mean value comparison

	Fontan						
	All patients	HLHS			Fontan Aortic status		
	(*n* = 20)	Yes (*n* = 14)	No (*n* = 6)		Non-reconstructed (*n* = 2)	Norwood (*n* = 18)	
cPWV	5.5 ± 1.2	5.3 ± 1.2	6.0 ± 1.1	*p* = 0.3	5.0 ± 0.9	5.6 ± 1.2	*p* < 0.001*
aPWV	7.2 ± 2.4	7.1 ± 2.5	7.3 ± 2.3	*p* = 0.9	6.2 ± 1.2	7.3 ± 2.5	*p* < 0.001*
	*p* = 0.009*	*p* = 0.03*	*p* = *0.2*		*p* = 0.005*	*p* = 0.01*	

	Controls			
	All patients	HLHS		
	(*n* = 49)	Yes (*n* = 5)	No (*n* = 44)	
cPWV	5.3 ± 1.0	5.0 ± 1.5	5.3 ± 0.9	*p* = *0.2*
aPWV	4.9 ± 0.7	4.8 ± 0.5	4.9 ± 0.7	*p* = *0.6*
	*p* = *0.1*	*p* = *0.7*	*p* = *0.08*	

Values in italics indicate statistical significance (*p* ≤ 0.05)

Mean value comparison between PWVs’ of Fontan- and Control-group. Values are given in m/s ± SD. *P* values were calculated from the two previous or preceding values using T-Test test for parametric and Wilcoxon-rank-sum-test test for non-parametric dependent samples

*aPWV* Pulse wave velocity in ascending aorta, *cPWV* Pulse wave velocity in entire central aorta, *HLHS* Hypoplastic left heart syndrome

Significant results (*p* < 0.05) are marked with *

We also conducted a sub-analysis where we compared our HLHS patients with the other FO patients (see Table 4). Firstly, the PWVs of the HLHS patients were not significantly different from those of the controls, and secondly, we found no significant differences between patients with and without HLHS.

Being aware that our sample size is too small to perform subgroup analysis (Norwood: *n* = 18, Non-reconstructed: *n* = 2), we found that Norwood patients had faster cPWV (Norwood: 5.6 ± 1.2 m/s, Non-reconstructed: 5.0 ± 0.9 m/s; *p* < 0.001) and aPWV (Norwood: 7.3 ± 2.5 m/s, Non-reconstructed: 6.2 ± 1.2 m/s; *p* < 0.001) in terms of aortic status.

There were also five HLHS patients in our controls, having received heart transplantation as young surgically untreated patients. Compared with the other transplanted patients, PWV was equal. Group specific sub-analysis are presented in Table 4.

### Haemodynamics

Comparing haemodynamics between groups, we found as expected that mean CVP was significantly higher in FO than in Controls (FO: 10.1 ± 3.1|Controls: 3.9 ± 2.8 mmHg; *p* < 0.001) and CI significantly reduced (FO: 3.7 ± 1.1|Controls: 4.5 ± 1.1 l/min/m^2^; *p* < 0.05). None of the other values differed significantly between groups (see Table 3; Fig. 1).

**Fig. 3:**
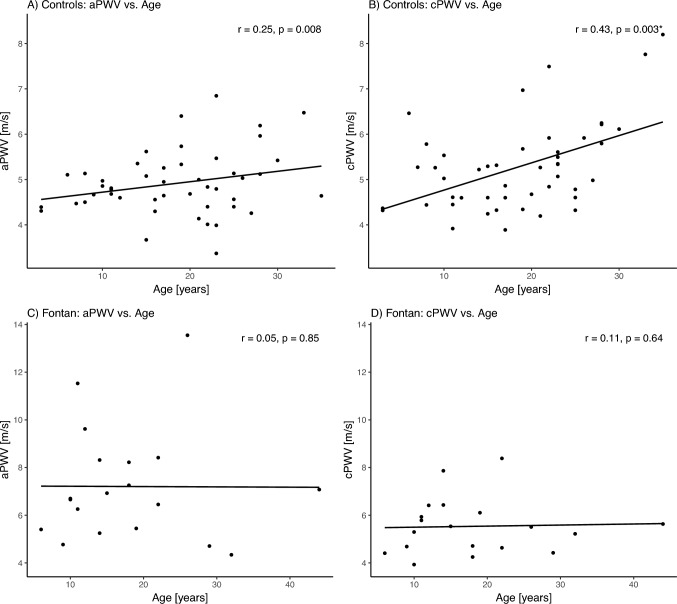
Scatter plots showing the relationship between aPWV/cPWV and age for both the Fontan and Control groups. *aPWV * pulse wave velocity of ascending aorta; *cPWV* pulse wave velocity of entire central aorta

We conducted a correlation analysis to determine how PWV is associated with the other parameters (see Table 5). Our Controls’ cPWV correlated moderately significantly with systolic (*r* = 0.42), diastolic (*r* = 0.48) and mean (*r* = 0.49) arterial blood pressures. All central pressures in the FO group correlated moderately with the cPWV, but this correlation did not reach significance. The aPWV revealed no association with the other measured haemodynamic parameters. We also identified a significant moderate increase in PWV in the Controls with age (*r* = 0.43) and SVRI (*r* = 0.44) and a decrease with CI (*r* = − 0.37). Figure 3 graphically illustrates the corresponding scatter plots of the correlation analysis between PWV and age.

**Table 5:** Correlation analysis

	Fontan				Controls			
	aPWV		cPWV		aPWV		cPWV	
Age	*r* = 0.05	(*p* = 0.85)	*r* = 0.11	(*p* = 0.64)	*r* = 0.25	(*p* = 0.08)	*r* = 0.43	(*p* = 0.003)*
Gender	*r* = − 0.08	(*p* = 0.75)	*r* = 0.35	(*p* = 0.15)	*r* = − 0.02	(*p* = 0.91)	*r* = − 0.11	(*p* = 0.45)
Systole	*r* = 0.17	(*p* = 0.50)	*r* = 0.31	(*p* = 0.22)	*r* = 0.08	(*p* = 0.62)	*r* = 0.42	(*p* = 0.01)*
Diastole	*r* = − 0.04	(*p* = 0.89)	*r* = 0.46	(*p* = 0.06)	*r* = 0.10	(*p* = 0.52)	*r* = 0.48	(*p* = 0.001)*
MAP	*r* = 0.04	(*p* = 0.88)	*r* = 0.37	(*p* = 0.13)	*r* = 0.11	(*p* = 0.49)	*r* = 0.49	(*p* = 0.0009)*
CVD	*r* = − 0.12	(*p* = 0.61)	*r* = − 0.006	(*p* = 0.98)	*r* = − 0.15	(*p* = 0.32)	*r* = − 0.14	(*p* = 0.37)
EDP	*r* = − 0.21	(*p* = 0.39)	*r* = − 0.28	(*p* = 0.25)	*r* = − 0.25	(*p* = 0.11)	*r* = − 0.23	(*p* = 0.14)
PCWP	*r* = − 0.07	(*p* = 0.81)	*r* = 0.006	(*p* = 0.9)	*r* = 0.02	(*p* = 0.96)	*r* = − 0.38	(*p* = 0.31)
CI	*r* = 0.09	(*p* = 0.75)	*r* = 0.06	(*p* = 0.82)	*r* = 0.005	(*p* = 0.98)	*r* = − 0.37	(*p* = 0.02)*
SVR	*r* = − 0.06	(*p* = 0.83)	*r* = − 0.13	(*p* = 0.67)	*r* = − 0.17	(*p* = 0.34	*r* = 0.21	(*p* = 0.24)
SVRI	*r* = 0.18	(*p* = 0.54)	*r* = 0.53	(*p* = 0.86)	*r* = − 0.11	(*p* = 0.55)	*r* = 0.44	(*p* = 0.008)*

Results of Pearson rank correlation between pulse wave velocity and central blood pressures in different measurement-positions with Pearson’s r and associated *p* values

*aPWV* Pulse wave velocity in ascending aorta, *cPWV* Pulse wave velocity in entire central aorta, *CI* cardiac pressure, *CVD* central venous pressure, *EDP* end-diastolic pressure, *MAP* mean arterial pressure, *PCWP* pulmonary capillary wedge pressure, *SVR* systemic vascular resistance, *SVRI* systemic vascular resistance index

*P* values were calculated using paired t-test for parametric and Wilcoxon-rank-sum test for non-parametric dependent samples

Significant results (*p* < 0.05) are marked with *

## Discussion

In this study, we conducted a detailed analysis of pulse wave velocity (PWV) in Fontan patients (FO), specifically focussing on PWV in the ascending aorta (aPWV) and the entire aorta up to iliac artery (cPWV). We compared these measurements with those from patients with biventricular physiology, including heart transplant recipients. FO presented a significantly higher aPWV (7.2 ± 2.4 m/s) than the Controls (4.9 ± 0.7 m/s). We also found that aPWV in the FO was higher than cPWV (5.5 ± 1.2 m/s) indicating a specifically stiffer ascending aorta. Interestingly, we observed no significant differences in aPWV or cPWV in the control group, nor did cPWV vary between the FO and controls.

As even minor distance measurement errors can result in significant deviations in PWV, we previously reported our interobserver variability [14]. Our findings suggest that our method is observer-independent with a mean derivation of 0.08 m/s for aPWV and 0.04 m/s for cPWV. The interobserver variability in our study closely matches that observed by Weber et al. [15, 16] who determined a mean deviation of 0.06 m/s. Previous research of Fontan patients’ PWV primarily relied on non-invasive methods like MRI, oscillometry or tonometry, and these studies often lacked comparisons with healthy control groups. In our study, on the other hand, we took an invasive approach to assess PWV in FO at two distinct aortic locations, allowing for meaningful comparisons with a control group with biventricular physiology. Our findings align with the results of Schäfer et al. [17], who observed increased PWV in the ascending aorta (aPWV) in FO, in particular attributable to patients with HLHS rather than other single ventricle congenital heart diseases. Non-compliant graft material, the presence of fibrous tissue, and aortic dilation are discussed as possible causative factors. To further refine our analysis, we conducted a subgroup comparison of HLHS patients with other Fontan entities on the one hand, but were unable to identify any significant PWV-differences while acknowledging the small number of patients (see Table 4). This suggests that the increased vascular stiffness in FO may not solely depend on this underlying single-ventricle condition. Kojima et al. [18] focussed on 56 paediatric single-ventricle patients (SiV) and detected a strong association between PWV and aortic dilatation. Compared to our PWVs, theirs were slower (4.83 ± 0.10 m/s) possibly attributable to different measurement locations (PWV of the entire intrathoracic aorta up to the diaphragm level) and younger age (about 3.5–4.5 years). As the present study lacked a comparison with healthy controls, the question as to whether Fontan patients generally have increased PWV cannot be answered.

Other studies showed that certain types of cardiac surgery such as the Norwood procedure could increase aortic stiffness [19–23]. Our results also indicated an increased aortic stiffness in FO, especially in those received aortic arch repair. The focus should therefore not only be on the underlying disease but also on the blood flow in the outflow tract. Manipulation in this area can lead to increased vascular stiffness [20, 24]. This is consistent with our difference between reconstructed and non-reconstructed aorta in FOs. Again, we are aware of the limited statistical significance within our small number of FO patients (see Table 4).

As can also be seen in Table 4, our controls included five patients with HLHS. All patients underwent heart transplantation in early childhood without prior surgical reconstruction. In these patients, PWV did not differ from other heart transplant patients. Nevertheless, our heart-transplant patients had undergone surgery involving aortic anastomosis as well.

The impact of heart transplantation on PWV is also multifaceted. While the immediate effects of successful transplantation may lead to decreased PWV thanks to improved cardiac output and alleviated heart failure symptoms, long-term factors such as immunosuppression and arterial remodelling can increase PWV. In addition, transplanted patients carry a higher risk for arterial hypertension [25], which further influences aortic stiffness [8, 26–28]. Nevertheless, PWV analysis did not reveal an increased PWV in those patients receiving blood pressure medication. As the influence on PWV cannot be quantified precisely, it should be lower in transplanted patients with their short and circular anastomoses, especially when compared to a potentially complex reconstructed aortic arch. In terms of age and BMI, our two groups are nearly equivalent (see Table 1). While that facilitates a sound comparison between groups, it is worth noting that FO patients are likely to be smaller [29].

Elmenhorst et al. [30] examined the pulse wave velocity in 1445 children and young adults to calculate percentiles. The percentiles are consistent with the PWV values of our patients, which indicates that the PWV is quite comparable to the PWV of a normal population. Therefore, women of the same average age had aortic PWV values between 4.5 and 5.0 m/s and men between 5.0 and 5.5 m/s (25th–75th percentile). In comparison, our controls’ aPWV was in the same range at 4.9 ± 0.6 m/s in females and 5.0 ± 0.8 m/s in males.

Age and haemodynamics also affect PWV and therefore vascular stiffness. We consistently observed a significant PWV-increase with age (see Table 5; Fig. 3). In line with disease-specific expectations [2, 5, 17, 31], we documented increased CVP and decreased CI within FO. Arterial blood pressure influences arterial stiffness [8, 26, 27] by determining the cardiac afterload and influencing the tension in the arterial vessel walls, which on the other hand is also attributable to vascular resistance. Consistent with physiologic expectations, we identified a significant moderate correlation between cPWV, central blood pressure, and SVRI in controls (see Table 5) and a non-significant but still moderate correlation within FO. To the best of our knowledge, few studies have demonstrated a relationship between SRV and PWV confirming Obata et al. [24] data. The aPWV we observed was not associated with other haemodynamic value measured in none of the groups. Since both groups had undergone aortic surgery of the proximal aortic parts (influencing distensibility and reservoir capacity), their blood pressure (preload on the aortic wall) might have less influence on their aPWV than possibly would in a native aortic arch.

## Strengths and Limitations

Our study is one of the few invasive investigations into pulse wave velocity (PWV) within a Fontan population, providing valuable data with a high degree of measurement precision, measuring PWV at two different sites, the “operated aortic arch” and entire aorta compared to a “biventricular” control group. However, our study must be interpreted acknowledging certain limitations. Regarding measurement errors dependent on the examiner, even minor inaccuracies, particularly in measurements on small children, can result in significant deviations. However, a previous study of ours indicated negligible interobserver variability. In relation to the study population, our group of Fontan patients is rather small, although it reveals standard high-quality clinical care entailing diagnostic imaging modalities such as MRI, making cardiac catheterisation in FO unnecessary for exclusively diagnostic purposes. The various underlying congenital heart diseases among a low number of FO patients, restricted our congenital heart disease-specific PWV assessment. Furthermore, our control group, although representing biventricular physiology, may not constitute vascularly healthy patients i.e. the fact of their having undergone aortic anastomosis surgery, may affect have affected aortic stiffness as well. Carrying out invasive catheterisation in healthy children for research purposes only is of course medically and ethically not justifiable.

## Conclusion

According to our exploratory study, Fontan patients reveal increased PWV in the aortic arch but not throughout the aorta. This finding may reflect specific aortic arch vascular stiffness rather than increased general aortic stiffness in this patient population. Although we can only speculate the reasons for our findings, our data may indicate surgical reconstruction might have an important impact. Further research is needed to understand the implications of these findings, to draw a possible conclusion such as the replacement of the reconstructed aortic arch when heart transplantation may be required in Fontan patients.

## Data Availability

The data underlying this article will be shared on reasonable request to the corresponding author.

## Abbreviations

aPWV: Pulse wave velocity in ascending aorta
CI: Cardiac index
CO: Cardiac output
cPWV: Pulse wave velocity in entire central aorta
CVP: Central venous pressure
DBP: Diastolic blood pressure
EDP: End-diastolic systemic ventricular pressure
FO: Fontan-patients
HLHS: Hypoplastic left heart syndrome
MAP: Mean arterial blood pressure
MP: Multipurpose catheter
PCWP: Pulmonary capillary wedge pressure
PWV: Pulse wave velocity
SBP: Systolic blood pressure
SiV: Single ventricular anatomy
SVR: Sytemic vascular resistance
SVRI: Sytemic vascular resistance index

## References

[1] Gordon-Walker TT, Bove K, Veldtman G (2019) Fontan-associated liver disease: a review. J Cardiol 74:223–232. 10.1016/j.jjcc.2019.02.016 30928109 10.1016/j.jjcc.2019.02.016

[2] Gewillig M, Brown SC (2016) The Fontan circulation after 45 years: update in physiology. Heart 102:1081–1086. 10.1136/heartjnl-2015-307467 27220691 10.1136/heartjnl-2015-307467 PMC4941188

[3] Sharma VJ, Iyengar AJ, Zannino D, Gentles T, Justo R, Celermajer DS, Bullock A, Winlaw D, Wheaton G, Burchill L, Cordina R, d’Udekem Y (2021) Protein-losing enteropathy and plastic bronchitis after the Fontan procedure. J Thorac Cardiovasc Surg 161:2158–2165. 10.1016/j.jtcvs.2020.07.107 32928546 10.1016/j.jtcvs.2020.07.107

[4] Rychik J, Atz AM, Celermajer DS, Deal BJ, Gatzoulis MA, Gewillig MH, Hsia T-Y, Hsu DT, Kovacs AH, McCrindle BW, Newburger JW, Pike NA, Rodefeld M, Rosenthal DN, Schumacher KR, Marino BS, Stout K, Veldtman G, Younoszai AK, d’Udekem Y (2019) Evaluation and management of the child and adult with Fontan circulation: a scientific statement from the American Heart Association. Circulation 140:234–284. 10.1161/CIR.0000000000000696 10.1161/CIR.0000000000000696 31256636

[5] Senzaki H, Masutani S, Ishido H, Taketazu M, Kobayashi T, Sasaki N, Asano H, Katogi T, Kyo S, Yokote Y (2006) Cardiac rest and reserve function in patients with Fontan circulation. J Am Coll Cardiol 47:2528–2535. 10.1016/j.jacc.2006.03.022 16781384 10.1016/j.jacc.2006.03.022

[6] Olivier M, O’Leary PW, Pankratz VS, Lohse CM, Walsh BE, Tajik AJ, Seward JB (2003) Serial Doppler assessment of diastolic function before and after the Fontan operation. J Am Soc Echocardiogr 16:1136–1143. 10.1067/s0894-7317(03)00635-7 14608284 10.1067/S0894-7317(03)00635-7

[7] Shachar GB, Fuhrman BP, Wang Y, Lucas RV, Lock JE (1982) Rest and exercise hemodynamics after the Fontan procedure. Circulation 65:1043–1048. 10.1161/01.cir.65.6.1043 7074766 10.1161/01.cir.65.6.1043

[8] Laurent S, Boutouyrie P, Lacolley P (2005) Structural and genetic bases of arterial stiffness. Hypertension 45:1050–1055. 10.1161/01.HYP.0000164580.39991.3d 15851625 10.1161/01.HYP.0000164580.39991.3d

[9] Laurent S, Cockcroft J, van Bortel L, Boutouyrie P, Giannattasio C, Hayoz D, Pannier B, Vlachopoulos C, Wilkinson I, Struijker-Boudier H (2006) Expert consensus document on arterial stiffness: methodological issues and clinical applications. Eur Heart J 27:2588–2605. 10.1093/eurheartj/ehl254 17000623 10.1093/eurheartj/ehl254

[10] O’Rourke M, Mancia G (1999) Arterial stiffness. J Hypertens 17:1–4. 10.1097/00004872-199917010-00001 10100086 10.1097/00004872-199917010-00001

[11] Zieman SJ, Melenovsky V, Kass DA (2005) Mechanisms, pathophysiology, and therapy of arterial stiffness. Arterioscler Thromb Vasc Biol 25:932–943. 10.1161/01.ATV.0000160548.78317.29 15731494 10.1161/01.ATV.0000160548.78317.29

[12] Myers KA, Leung MT, Terri Potts M, Potts JE, Sandor GGS (2013) Noninvasive assessment of vascular function and hydraulic power and efficiency in pediatric Fontan patients. J Am Soc Echocardiogr 26:1221–1227. 10.1016/j.echo.2013.06.013 23860097 10.1016/j.echo.2013.06.013

[13] Sarkola T, Jaeggi E, Slorach C, Hui W, Bradley T, Redington AN (2013) Assessment of vascular remodeling after the Fontan procedure using a novel very high resolution ultrasound method: arterial wall thinning and venous thickening in late follow-up. Heart Vessels 28:66–75. 10.1007/s00380-011-0217-2 22331173 10.1007/s00380-011-0217-2

[14] Walser M, Schlichtiger J, Dalla-Pozza R, Mandilaras G, Tengler A, Ulrich S, Oberhoffer FS, Oberhoffer-Fritz R, Böhm B, Haas NA, Jakob A (2023) Oscillometric pulse wave velocity estimated via the Mobil-O-Graph shows excellent accuracy in children, adolescents and young adults: an invasive validation study. J Hypertens 41:597–607. 10.1097/HJH.0000000000003374 36723480 10.1097/HJH.0000000000003374

[15] Hametner B, Wassertheurer S, Kropf J, Mayer C, Eber B, Weber T (2013) Oscillometric estimation of aortic pulse wave velocity: comparison with intra-aortic catheter measurements. Blood Press Monit 18:173–176. 10.1097/MBP.0b013e3283614168 23571229 10.1097/MBP.0b013e3283614168

[16] Weber T, Maas R, Auer J, Lamm G, Lassnig E, Rammer M, O’Rourke MF, Böger RH, Eber B (2007) Arterial wave reflections and determinants of endothelial function a hypothesis based on peripheral mode of action. Am J Hypertens 20:256–262. 10.1016/j.amjhyper.2006.09.009 17324736 10.1016/j.amjhyper.2006.09.009

[17] Schäfer M, Younoszai A, Truong U, Browne LP, Mitchell MB, Jaggers J, Campbell DN, Hunter KS, Ivy DD, Di Maria MV (2019) Influence of aortic stiffness on ventricular function in patients with Fontan circulation. J Thorac Cardiovasc Surg 157:699–707. 10.1016/j.jtcvs.2018.09.039 30396734 10.1016/j.jtcvs.2018.09.039

[18] Kojima T, Kuwata S, Kurishima C, Iwamoto Y, Saiki H, Ishido H, Masutani S, Senzaki H (2014) Aortic root dilatation and aortic stiffness in patients with single ventricular circulation. Circ J 78:2507–2511. 10.1253/circj.cj-13-1264 25109427 10.1253/circj.cj-13-1264

[19] Biglino G, Schievano S, Steeden JA, Ntsinjana H, Baker C, Khambadkone S, de Leval MR, Hsia TY, Taylor AM, Giardini A (2012) Reduced ascending aorta distensibility relates to adverse ventricular mechanics in patients with hypoplastic left heart syndrome: noninvasive study using wave intensity analysis. J Thorac Cardiovasc Surg. 10.1016/j.jtcvs.2012.08.028 10.1016/j.jtcvs.2012.08.028 23031685

[20] Biko DM, Gaynor JW, Partington SL, Harris MA, Whitehead KK, Trusty P, Yoganathan AP, Fogel MA (2019) Relationship of aortic stiffness to exercise and ventricular volumes in single ventricles. Ann Thorac Surg 108:574–580. 10.1016/j.athoracsur.2019.03.019 30959013 10.1016/j.athoracsur.2019.03.019

[21] Cardis BM, Fyfe DA, Mahle AT (2006) Elastic properties of the reconstructed aorta in hypoplastic left heart syndrome. Ann Thorac Surg 81:988–991. 10.1016/j.athoracsur.2005.09.065 16488707 10.1016/j.athoracsur.2005.09.065

[22] Kim GB, Kang SJ, Bae EJ, Yun YS, Noh CI, Lee JR, Kim YJ, Lee JY (2004) Elastic properties of the ascending aorta in young children after successful coarctoplasty in infancy. Int J Cardiol 97:471–477. 10.1016/j.ijcard.2003.10.020 15561335 10.1016/j.ijcard.2003.10.020

[23] Ong CM, Canter CE, Gutierrez FR, Sekarski DR, Goldring DR (1992) Increased stiffness and persistent narrowing of the aorta after successful repair of coarctation of the aorta: relationship to left ventricular mass and blood pressure at rest and with exercise. Am Heart J 123:1594–1600. 10.1016/0002-8703(92)90815-d 1595541 10.1016/0002-8703(92)90815-d

[24] Obata Y, Mizogami M, Singh S, Nyhan D, Berkowitz DE, Steppan J, Barodka V (2016) The effects of hemodynamic changes on pulse wave velocity in cardiothoracic surgical patients. Biomed Res Int 2016:1–7. 10.1155/2016/9640457 10.1155/2016/9640457 PMC5120184 27900333

[25] Bansal N, Raedi WA, Medar SS, Abraham L, Beddows K, Hsu DT, Lamour JM, Mahgerefteh J (2023) Masked hypertension in pediatric heart transplant recipients. Pediatr Cardiol. 10.1007/s00246-023-03096-y 10.1007/s00246-023-03096-y 36656319

[26] Mitchell GF (2014) Arterial stiffness and hypertension: chicken or egg? Hypertension 64:210–214. 10.1161/HYPERTENSIONAHA.114.03449 24799614 10.1161/HYPERTENSIONAHA.114.03449 PMC4185002

[27] Roman MJ, Devereux RB, Kizer JR, Lee ET, Galloway JM, Ali T, Umans JG, Howard BV (2007) Central pressure more strongly relates to vascular disease and outcome than does brachial pressure: the Strong Heart Study. Hypertension 50:197–203. 10.1161/HYPERTENSIONAHA.107.089078 17485598 10.1161/HYPERTENSIONAHA.107.089078

[28] Nilsson PM, Khalili P, Franklin SS (2014) Blood pressure and pulse wave velocity as metrics for evaluating pathologic ageing of the cardiovascular system. Blood Press 23:17–30. 10.3109/08037051.2013.796142 23750722 10.3109/08037051.2013.796142

[29] Mancilla EE, Zielonka B, Roizen JD, Dodds KM, Rand EB, Heimall JR, Chen F, Wu C, Goldberg DJ, Rychik J (2021) Growth in children with a Fontan circulation. J Pediatr 235:149–155. 10.1016/j.jpeds.2021.04.019 33887332 10.1016/j.jpeds.2021.04.019

[30] Elmenhorst J, Hulpke-Wette M, Barta C, Dalla Pozza R, Springer S, Oberhoffer R (2015) Percentiles for central blood pressure and pulse wave velocity in children and adolescents recorded with an oscillometric device. Atherosclerosis 238:9–16. 10.1016/j.atherosclerosis.2014.11.005 25461733 10.1016/j.atherosclerosis.2014.11.005

[31] Ohuchi H, Miyazaki A, Negishi J, Hayama Y, Nakai M, Nishimura K, Ichikawa H, Shiraishi I, Yamada O (2017) Hemodynamic determinants of mortality after Fontan operation. Am Heart J 189:9–18. 10.1016/j.ahj.2017.03.020 28625386 10.1016/j.ahj.2017.03.020

